# Progress in the Research on Branched Polymers with Emphasis on the Chinese Petrochemical Industry

**DOI:** 10.3390/molecules28237934

**Published:** 2023-12-04

**Authors:** Yi Pan, Jie Bai, Gang Yang, Zhaoxuan Li

**Affiliations:** 1 Department of Petroleum and Natural Gas Engineering College, Liaoning Petrochemical University, No. 1, West Section of Dandong Road, Wanghua District, Fushun 113001, China; panyi_bj@126.com (Y.P.); bai_jie123@163.com (J.B.); 2 Sludge Department of China National Petroleum Corporation, Greatwall Well Drilling Company, Panjin 124000, China; yang_1230411@163.com

**Keywords:** polymer flooding, branched polymer, hyperbranched macromolecules, oil displacement agent, petrochemical industry, application

## Abstract

Polymer flooding, one of the main methods for improving crude oil recovery using chemical flooding technology in China, is widely used for actual oil displacement. Partially hydrolyzed polyacrylamide (HPAM) is a commonly used linear polymer in polymer flooding, but it exhibits poor temperature and salt resistance due to its molecular structure. Therefore, branched polymers have been studied. This article provides a review of the specific synthetic methods and current practical applications in the petrochemical field of dendritic polymers and hyperbranched macromolecules. The advantages and disadvantages of each synthetic method for branched polymers are also elaborated. Finally, the application prospects of branched polymers are discussed. The feasibility of branched polymers in large quantities should be further verified through additional field tests, which should address concerns such as synthesis costs and reaction efficiency.

## 1. Introduction

At the onset of 2022, the Economic and Technological Research Institute of China Petroleum Corporation unveiled the “Development Report on the Domestic and Foreign Oil and Gas Industry”. The report highlights that in 2021, China’s investment in oil exploration and development continued to surge, resulting in sustained growth in both oil production and reserves. Crude oil production witnessed a commendable increase of over 2%, reaching an impressive 199 million tons. Forecasts indicate that domestic oil exploration and development will maintain its accelerated pace throughout 2022. Recently, the National Energy Administration issued the “2022 Energy Work Guidance”, which sets a specific target for crude oil production at approximately 200 million tons [1]. However, it is worth noting that China’s primary oil fields are currently undergoing water injection development in their intermediate–late stages. Unfortunately, this process often leads to injected water flowing along high-permeability layers while bypassing areas with abundant oil resources, ultimately leaving significant amounts of untapped crude oil behind. Consequently, this inefficient water drive recovery approach results in a crude oil recovery rate below 40%. The proportion of China’s crude oil imports is projected to surpass 70% by 2021 [2]. Therefore, enhancing crude oil recovery effectiveness has consistently remained one of the pivotal concerns in petrochemical research.

Polymer flooding has consistently been among the primary strategies for enhancing crude oil recovery due to its uncomplicated materials, economical cost, and ability to significantly enhance the water-phase mobility ratio and substantially increase revenue. Field trials of polymer flooding have been conducted at the Byron Oilfield in the North Oregon Basin, Wyoming; the Regular Oilfield and the Twin Peaks Oilfield in Texas; and the Marmul Oilfield in Oman [3]. The experimental results indicate that the application of polymer flooding technology can boost crude oil recovery by 6% to 17%. To date, the geological reserves covered by polymer flooding in China have exceeded one billion tons, with the annual production of polymer flooding remaining consistently at the level of ten thousand tons for many consecutive years. The average recovery rate of application blocks has increased by 12% [4,5]. Linear polyacrylamide (HPAM) is currently the primary polymer employed for oil displacement. It demonstrates excellent solubility and a short dissolution time in freshwater or low-salinity injected water. Upon dissolution, it benefits from the hydration layer film on the macromolecular chain and the electrostatic repulsion of carboxyl groups (-COO^−^) on the macromolecular skeleton, which confers superior viscosity enhancement and rheological properties [6]. This can effectively augment the crude oil recovery rate. Polymers with unique properties, such as temperature resistance and salt resistance, have been synthesized by Qi Shulei et al. [7]. Their instant polyacrylamide has been successfully implemented in the Gudao Oilfield, resulting in a significant increase in oil production and a final recovery rate of 63.6%. Zhou et al. [8] developed quaternary copolymers using functional monomers, including AM and AMPS. After aging in a solution with a salinity of 15,000 mg/L for 30 days, their viscosity retention rate reached 72.7%, and the average recovery rate of the polymer at a concentration of 1000 mg/L was 10.8%. However, in certain extreme environments with high temperatures and high mineralization, the presence of electrostatic shielding causes HPAM molecules to adopt a curly state, significantly reducing their tackiness. This leads to significant hydrolysis and thermal decomposition, resulting in poor salt and temperature resistance, and greatly diminishing their revenue enhancement effect. Consequently, ongoing research by scientists has led to the extensive modification of branched polymers with branched structures and terminal functional groups, thereby achieving high fluidity, low viscosity, and excellent solubility. The presence of side chains in branched polymer molecules mitigates chain curling, enhances molecular chain rigidity, and increases solution viscosity under high-temperature and high-salt conditions. In the polymer-shearing process, the branched polymer main chain is safeguarded by the side chain, resulting in superior shear resistance compared with HPAM [9,10]. The remarkable performance of branched polymers in complex oil reservoir environments has garnered significant attention from scholars, boasting extensive application prospects in the petrochemical industry. However, the published reviews lack specific evaluations regarding the classification of branched polymers and their associated applications.

**Figure 1:**
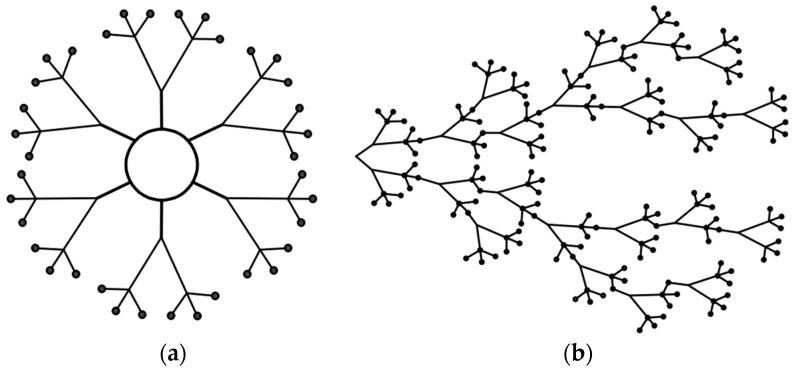
Structural diagrams of branched polymers: (**a**) dendritic polymer; (**b**) hyperbranched polymer.

The present paper provides a comprehensive review of the classification of branched polymers into dendrimers and hyperbranched macromolecules, their synthetic methods, as well as their respective advantages and disadvantages. Furthermore, it summarizes the recent advancements in the application of branched polymers within the petrochemical industry and proposes future research directions to serve as a valuable reference for related studies. Figure 1 shows the structure comparison between a dendritic polymer and a hyperbranched polymer.

## 2. Synthesis of Branched Polymers

**Figure 2:**
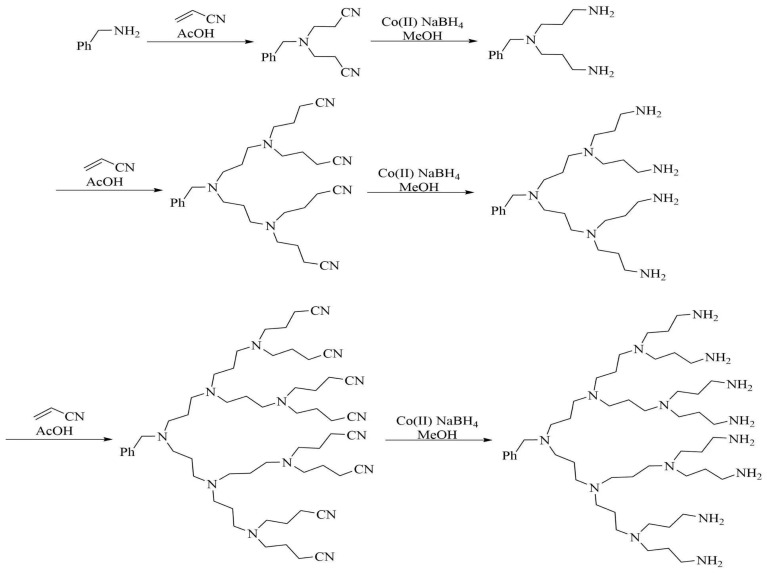
PPI synthetic route.

As early as 1952, American scholar Flory [11] investigated the potential of synthesizing highly branched macromolecules from multifunctional monomers. However, this research did not garner significant attention at the time. It was not until 1978 that Fritz Vogtle at the University of Bonn [12] first attempted to synthesize branched molecules utilizing the Michael addition reaction. By connecting three acrylonitrile groups to a nitrogen atom in an amino group, followed by reducing the nitrile group to primary ammonia and repeating the Michael addition reaction, Vogtle successfully obtained PPI small molecules. This innovative small molecule doubles the number of branched structural units per reaction generation, ultimately forming branched large molecules akin to branch structures in the fifth generation. Figure 2 shows the synthetic route of the PPI.

**Figure 3:**
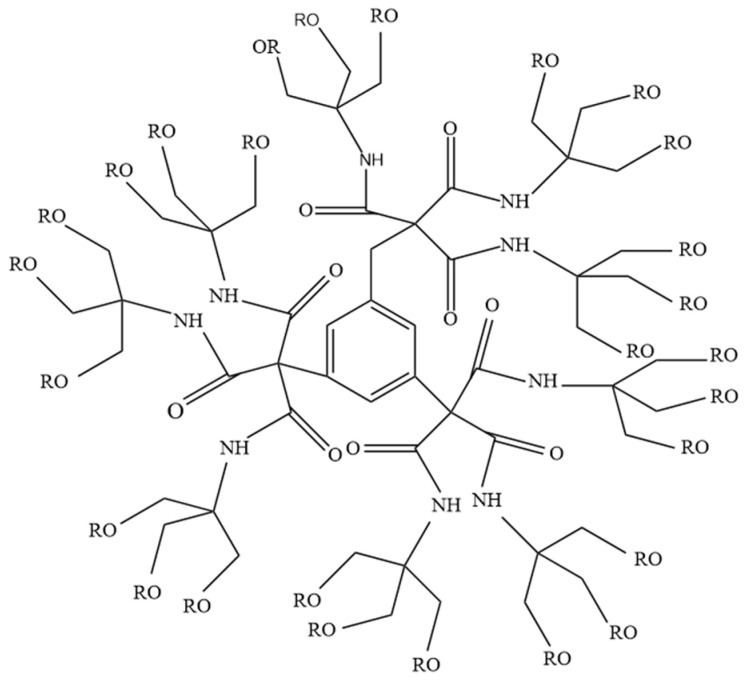
Schematic diagram of the molecular structure of arborols.

It was not until 1985, however, that the investigations by American researchers Tomalia and Newkome effectively integrated dendrimers into the realm of scientific research. Tomalia [13] successfully synthesized polyamidoamine (PAMAM) with a dendritic structure, branding it as “Startburst dendrimers”, and it is now primed for industrial production. In the same year, Newkome [14] also achieved the synthesis of a dendrimer with a trimethylbenzene ring as its core, branching outward, which he christened “arborols”, as illustrated in Figure 3. Consequently, a profusion of dendritic polymers with diverse materials, structures, and synthetic methods has emerged.

Polymers with a dendritic structure possess a highly branched architecture, enabling extensive modification of the functional groups at their terminals. This results in outstanding characteristics, such as high fluidity, low viscosity, and excellent solubility [15], which make these materials exceptionally suitable for applications in supramolecular chemistry, nanomaterials, catalysts, and petrochemicals.

**Figure 4:**
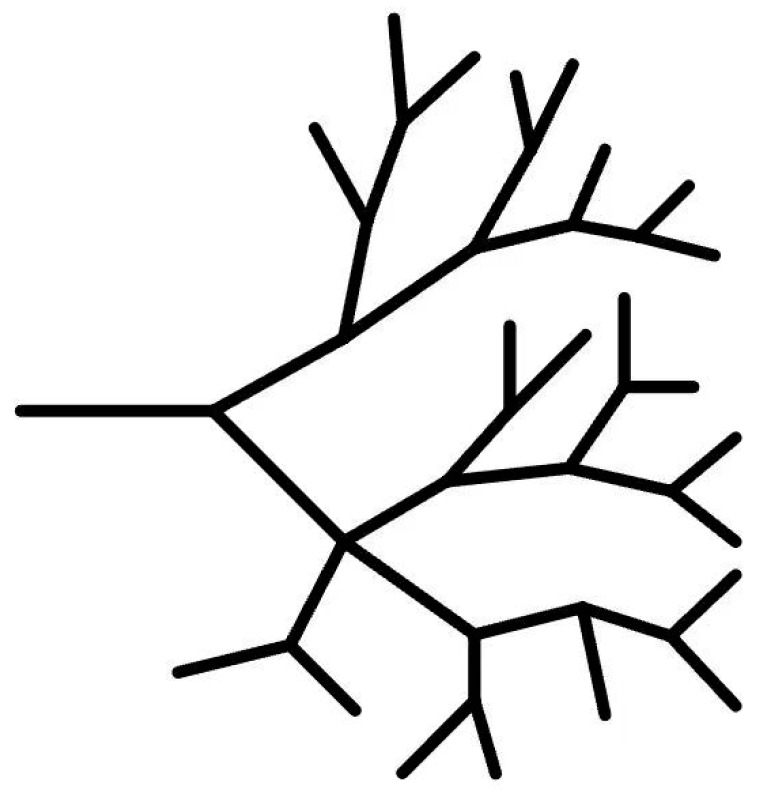
Schematic diagram of molecular structure of hyperbranched polymers.

Nonetheless, the highly symmetrical structure of dendritic polymers often necessitates numerous labor-intensive purification processes during synthesis, resulting in increased synthesis costs and limited production fault tolerance. Numerous unfavorable factors have impeded the advancement of dendritic polymer research [16]. Subsequently, it has been recognized that such an idealized symmetrical structure is not always essential. Consequently, hyperbranched polymers, which inherit the majority of dendritic polymers’ advantages despite possessing less symmetrical structures and relaxed synthetic conditions, have emerged in the scientific community’s field of vision. Figure 4 illustrates the molecular structure of a hyperbranched polymer.

The degree of branching (DB) is the physical quantity that characterizes the structural attributes of branched polymers [17]. It serves as a direct indicator of the structural density of a polymer, as well as the position and quantity of end functional groups [18]. The computation formula is as follows:

(1) $$\mathrm{DB}=\frac{D+T}{D+T+L}$$

D denotes the fully branched structural unit; T denotes the end structural unit; and L denotes the linear structural unit.

The formula dictates that when a branched polymer structure lacks linear structural units, the degree of branching (DB) is equivalent to one, indicating a structurally perfectly symmetrical polymer, i.e., a dendritic polymer. Nonetheless, hyperbranched macromolecules possess certain linear structural units, resulting in a DB value of less than one [19].

Polymers with branching structures can be categorized into two types: dendritic polymers, which lack linear structural units (DB value = 1), and hyperbranched polymers, which possess linear structural units (DB value of <1).

### 2.1. Dendritic Polymer

Polymers with dendritic structures, resulting from the iterative reactions between multifunctional monomers, are referred to as dendritic polymers. The three-dimensional structure of these polymer molecules can be distinctly divided into three components: the initial initiation core, the inner layer composed of multiple repeating units, and the outer surface layer with multiple functional groups [20].

Following an extensive body of research by scientists globally, the synthetic methods of dendritic polymers are currently primarily categorized into three types: divergent synthesis, convergent synthesis, and a combination of divergent and convergent approaches.

**Figure 5:**
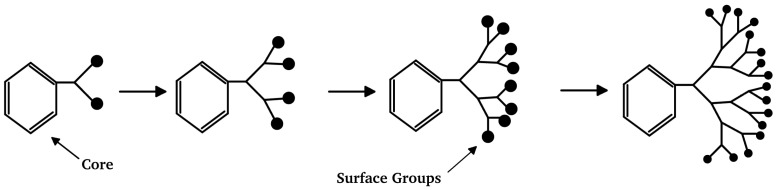
Schematic diagram of the principle of divergent synthesis.

Divergent synthesis, initially proposed by Vogtle and Buhleier [12], was further developed by Tomalia and Newkome [14,21]. This synthetic approach involves the initiation of polymerization steps progressively from the core of an initial core possessing a reactive functional group, gradually expanding the reaction sequence, and ultimately synthesizing a dendritic polymer. Figure 5 shows the schematic diagram of the divergent synthetic method.

**Figure 6:**
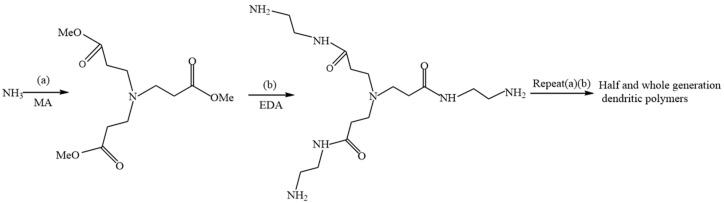
Synthetic route of dendritic polymer with ammonia as core: (a) ammonia and methyl acrylate (MA) by Michael addition reaction to form ternary ester (0.5 G); (b) ternary ester reacts with excess ammonia to form ternary amide compound (1.0 G).

Polymers with a branched structure were synthesized by Li Jie et al. [22] using a divergent synthetic method that employed ethylenediamine as the initiator core. Initially, a Michael addition reaction was conducted to react ethylenediamine with methyl acrylate, resulting in the formation of a quaternary ester (a half-generation 0.5 G reaction product). Subsequently, the quaternary ester underwent an ammonolysis reaction with excess ethylenediamine to generate a quaternary ester amine complex (a first-generation whole-generation reaction product). Finally, the aforementioned Michael addition and ammonolysis reactions were reiterated to produce PAMAM dendrimers of varying generations [23]. Figure 6 shows the synthetic route of a dendritic polymer with ammonia as its core.

**Figure 7:**
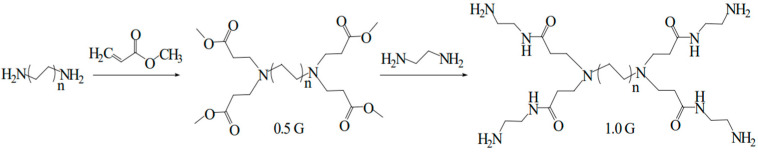
Schematic diagram of the synthetic route of 0.5 G and 1.0 G dendritic macromolecules.

Polymers with diverse structures were synthesized by Zhang Yu at Northeast Petroleum University [24] using a combination of ethylenediamine, hexanediamine, butanediamine, octanediamine, and methyl acrylate via Michael addition reactions. This approach generated a series of 0.5 G dendritic polymers. Subsequently, these dendritic polymers underwent ester amine condensation reactions with ethylenediamine, leading to the production of a range of 1.0 G dendritic polymers. Figure 7 shows the synthetic routes of 0.5 G and 1.0 G dendritic macromolecules.

To date, an increasing body of evidence demonstrates the viability of synthesizing dendritic polymers via divergent synthesis. Nonetheless, this method is concurrently characterized by a drawback: as the reaction algebra expands, the number of peripheral functional groups geometrically multiplies, leading to an exacerbation of steric hindrance effects. This, in turn, predisposes the dendritic molecular structure to defects and necessitates more stringent separation conditions for the products. Consequently, an alternative synthetic approach, referred to as convergent synthesis, has been proposed.

**Figure 8:**
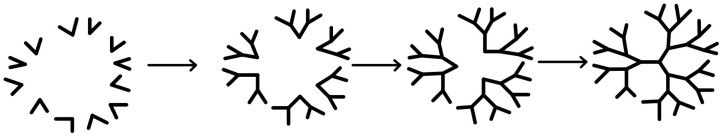
Schematic diagram of convergent synthetic route.

Contrary to the principle of divergent synthesis, which initially defines the central core and subsequently synthesizes peripheral functional groups, convergent synthesis initially consolidates the branches into larger branched structures, followed by a reaction with the synthesized symmetrical branched macromolecular units to generate a comprehensive dendritic polymer [25]. Figure 8 shows the schematic diagram of the synthetic route via convergent synthesis.

Frechet et al., from Cornell University [26], proposed the convergent synthetic approach. They reacted two molecules of benzyl bromide and its derivatives with one molecule of 3,5-dihydroxybenzyl alcohol, replacing the remaining hydroxyl groups with bromine atoms. This process was reiterated to obtain a fan-shaped polyphenylene ether. By selecting a multi-arm core and connecting it to the fan-shaped structure, a dendritic polyphenylene ether polymer with meta aryl branching and an ether connection was ultimately achieved.

The following advantages are observed when comparing this method with the divergent synthetic approach: ① decreased total synthesis steps; ② a simplified purification process for each step of the product, facilitating the obtainment of higher-purity products in the synthesis of higher-generation macromolecules; and ③ a reduced propensity of macromolecular surfaces for defects, enabling better control over the structure of dendritic polymers. Nonetheless, the convergent synthetic method is not devoid of drawbacks. Owing to the spatial divergence of higher-algebra branches and their pronounced steric hindrance effects, this method exhibits a low yield and is currently less prevalent in practice.

In addition, there is the divergent–convergent co-use approach proposed by Kawaguchi [27], which ingeniously integrates divergent synthesis and convergent synthesis to enhance yield by mitigating spatial hindrance effects. The process starts with the synthesis of a branched small molecule with low reaction algebra using the divergent method, which serves as the core. This is followed by the synthesis of a branched unit utilizing convergent synthesis and its connection to the core of the small molecule to form a dendritic macromolecule. By separately synthesizing the core and branching units while harnessing the benefits of both the divergent and convergent methods, it is possible to improve the yields of dendritic polymers, double the molecular weights, and facilitate purification processes during synthesis, ultimately enabling highly symmetrical dendritic polymer synthesis.

**Figure 9:**
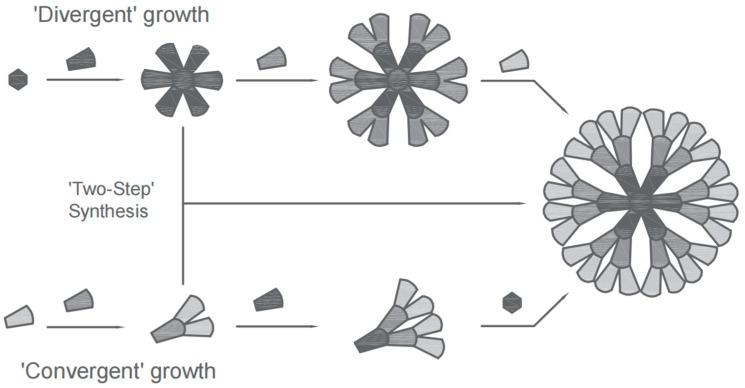
Schematic diagram of the route for synthesizing dendritic polymers using divergent–convergent co-use.

Despite the numerous merits of this approach, the challenges pertaining to labor-intensive reaction processes, elevated production costs, and the current dearth of research must be recognized, which have also impeded the method’s widespread implementation. Figure 9 shows the road map of the synthesis of a dendritic polymer using the divergent–convergent co-use method.

### 2.2. Hyperbranched Polymer

The origin of hyperbranched macromolecules can be traced back to the late 19th century. In the early stages of research, hyperbranched resins were synthesized via the condensation polymerization of organic acids and alcohols [28], but this structure remained unnamed. It was not until 1952 that Flory [11] initially introduced the concept of hyperbranched polymers. Subsequently, in 1992, Kim and Webster [29] successfully synthesized hyperbranched polystyrene, which, thereafter, garnered attention due to its distinctive properties.

**Figure 10:**
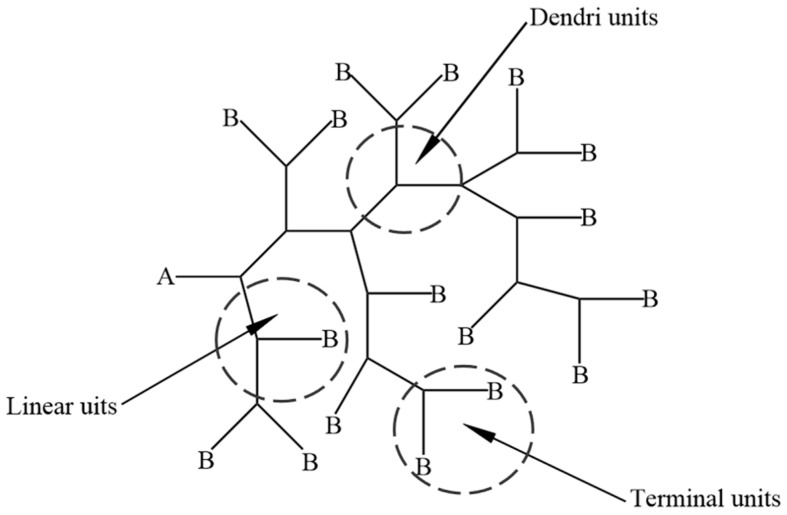
Schematic representation of recurring structural units in a hyperbranched polymer.

The hyperbranched polymer structure comprises three repeating units: a dendritic unit, a linear unit, and an end group consisting of an unreacted functional group B. In addition to the recurring structural units, it also features a central core A that is progressively extended by the branched monomer ABn to form the hyperbranched polymer, as illustrated in Figure 10.

In-depth exploration by scientists has led to the identification of several commonly employed synthetic methods for hyperbranched polymers. These methods include polycondensation synthesis, ring-opening polymerization, and free-radical polymerization.

#### 2.2.1. Polycondensation Synthesis

The method currently employed for the synthesis of hyperbranched macromolecules is both widely used and mature. Polycondensation refers to the reiterative process of condensation reactions between monomers possessing two or more active groups, resulting in the formation of polymers while liberating water and other small molecules. The majority of polycondensation reactions are reversible and gradual, characterized by a gradual increase in molecular weight with the extension of the reaction time. The reactants commonly used in polycondensation reactions possess ABx-type structures, rendering the reaction relatively straightforward. Upon the addition of catalysts and initiators to the solution in accordance with the established process flow, the polycondensation reaction can be conducted at elevated temperatures to yield hyperbranched polymers [30].

**Figure 11:**
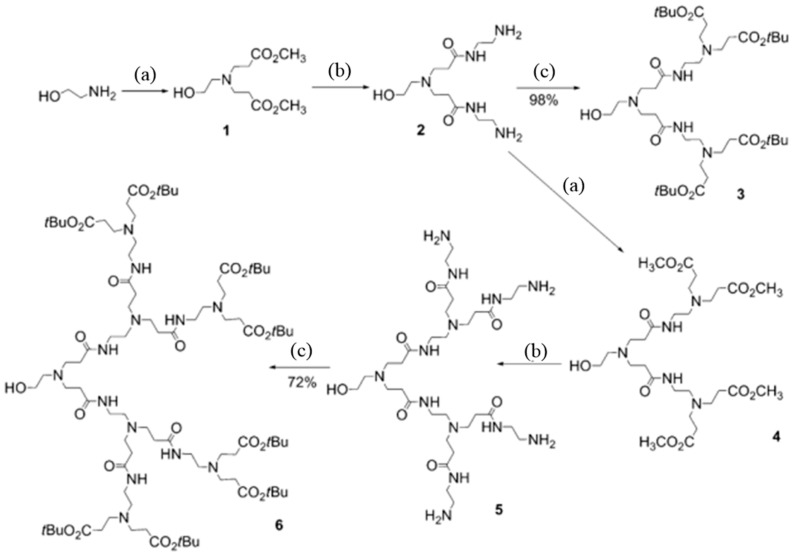
Synthesis of dendrons 1–6. Reagents and conditions: (a) methyl acrylate, MeOH, RT, and 3 days; (b) ethylenediamine, MeOH, 80 °C to RT, and 3 days; and (c) *tert*-butyl acrylate, MeOH, RT, and 3 days.

Rousseau et al. [31] synthesized hyperbranched polymers utilizing ethanolamine and methyl acrylate as raw materials via a Michael addition condensation reaction of carboxylic esters and amino groups. The reaction process is illustrated in Figure 10. This hyperbranched polymer exhibits advantages such as a high yield and mild reaction conditions. However, the reaction necessitates multiple repetitive operations, along with the requirement of purification for each step of the product before proceeding to the next, rendering the synthesis costly and inadequate for industrial applications. Furthermore, the reaction product contains a substantial amount of ester groups, which are susceptible to hydrolysis under high-temperature conditions, making it inappropriate for use in high-temperature environments. Figure 11 shows the route of AB2-type monomer polycondensation to synthesize hyperbranched polymers. The targeted PAMAM dendrons 3 and 6 have a hydroxyl moiety at their focal point, and peripheral *tert*-butyl ester groups were synthesized by reacting *tert*-butyl acrylate with amine-terminated PAMAM dendrons 2 and 5.

Wang et al. [32] synthesized terminal carboxyl hyperbranched polyesteramide (HBPEA) by employing ethanolamine (EMA) and benzene-1,2,4-tricarboxylic acid-1,2-anhydride (BTAA) as raw materials, employing both two-step and one-step melt polycondensation techniques. The highly reactive anhydride groups present in BTAA engage in amino reactions with EMA, generating AB2 intermediates containing two carboxyl groups and one hydroxyl group. Subsequently, the hyperbranched polyester amide is obtained via condensation polymerization, exhibiting properties akin to polycarbonate (PC).

**Figure 12:**
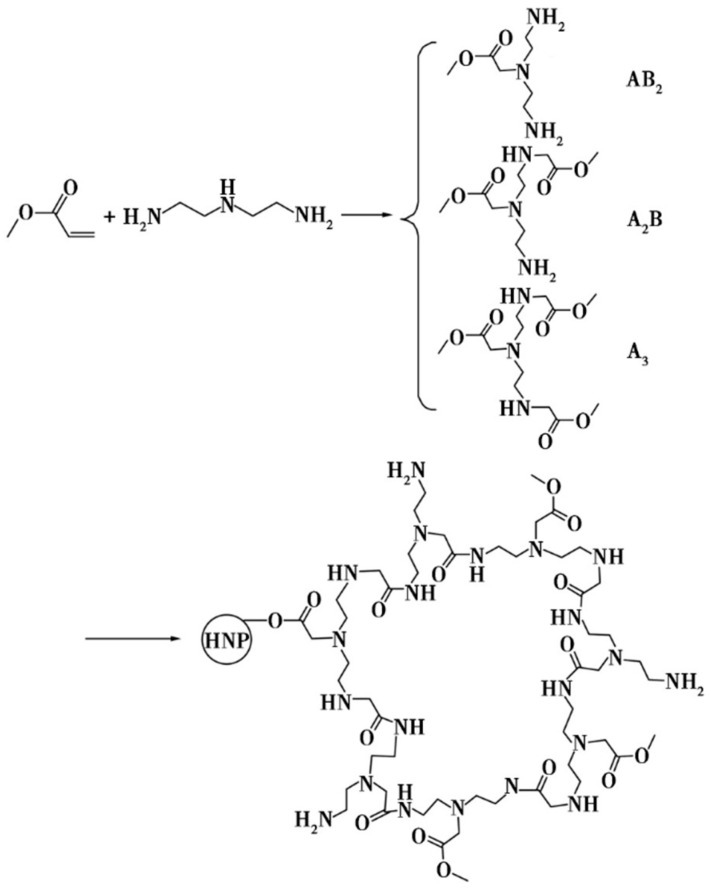
Synthetic reaction formula of carboxyl-terminated hyperbranched polymer.

The synthesis of the hyperbranched polyamide matrix HBP-NH2 by Qian Kai et al. [33] was achieved via the ABx-type monomer self-condensation method using methyl acrylate, diethylenetriamine, and methanol as raw materials. Subsequently, HBP-NH2 was modified with sodium acrylate to terminate the amino group, resulting in the synthesis of a carboxyl-terminated hyperbranched polymer, HBP-COOH, which exhibits scale-inhibitory properties. The corresponding reaction scheme is shown in Figure 12.

Polycondensation synthesis presents dispersibility challenges and offers limited control over the quality of the synthesized molecules, while the preparation process for ABx-type monomers involved in the reaction is intricate and resource-intensive. Nonetheless, due to its simplified synthesis, the elimination of the need for further separation and purification, and its capacity to preserve the physical and chemical properties of hyperbranched macromolecules, polycondensation has remained a crucial method for scientists to synthesize hyperbranched polymers to date.

#### 2.2.2. Ring-Opening Polymerization

The process of transforming cyclic compound monomers into linear polymers using ring-opening addition is denominated as ring-opening polymerization. The method employs heterocyclic compounds, including ethylene oxide, caprolactone, tetrahydrofuran, and cyclic carbamate, as the monomers for synthesizing hyperbranched polymers.

The synthesis of hyperbranched polyamines via the ring-opening polymerization of cyclic carbamates, via the catalysis of palladium, was initially reported by Suzuki et al. in 1992 [34]. This method was revisited by the same research group in 1998, employing 5-methylene-1,3-oxazolin-2-one as the raw material for the synthesis of hyperbranched polyamines.

Testud et al. [35] synthesized AB2 and AB3 monomers, which incorporated methyl ester and alcohol functional groups, via epoxidation utilizing sunflower, castor, and rapeseed oils as raw materials. Subsequently, hyperbranched polyesters were fabricated via monomer condensation and ester exchange reactions. Ultimately, polymer stability tests demonstrated that the hyperbranched polymer retained good stability above 300 °C.

**Figure 13:**
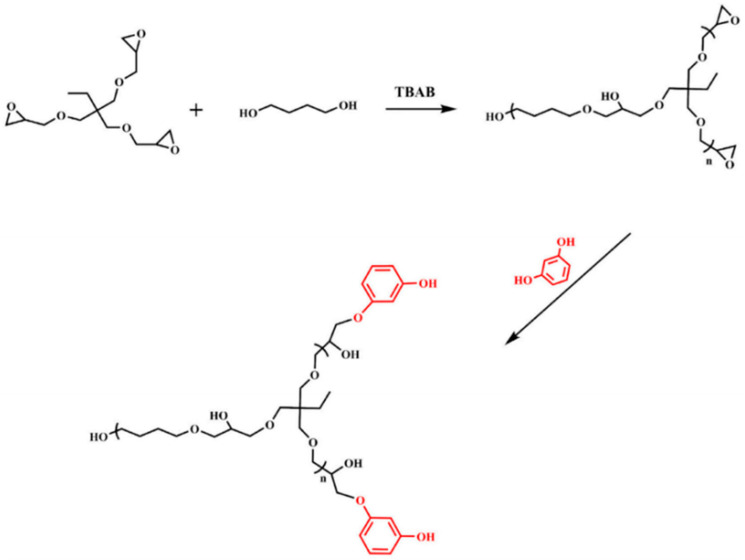
Synthetic route of hyperbranched polymers containing hydroxyl groups.

Polymers containing hydroxyl groups with tetrabutylammonium bromide (TBAB) as a phase-transfer catalyst and trimethylpropane triglycidyl ether, butanediol, and ortho diphenol as raw materials were synthesized by Xiao et al. [36] via ring-opening polymerization and condensation reactions, respectively. The synthetic route is shown in Figure 13.

Yang et al. [37] synthesized hydroxyl-terminated aromatic hyperbranched polyesters via ring-opening polymerization using trimethylolpropane as the central core, epichlorohydrin, and trimellitic anhydride. Thermal weight loss analysis was performed, revealing that there was no significant weight loss in the product prior to 120 °C. However, at 232 °C, the polymer main chain fractured, leading to a 94% weight loss in the resin. This finding suggests that the hyperbranched epoxy resin polymer does not contain small-molecule substances and exhibits excellent thermal stability.

#### 2.2.3. Free-Radical Polymerization

The monomers employed in free-radical polymerization are typically olefins possessing unsaturated double bonds. Throughout the reaction process, the double bonds in these monomers are cleaved, facilitating multiple addition reactions between molecules, ultimately leading to the formation of hyperbranched polymers. This technique was initially reported by Frechet et al. [38], and the monomers utilized ordinarily incorporate both a vinyl group and a reactive group capable of initiating vinyl polymerization. These reactive groups are generally represented by free radicals, cations, or anions. During the reaction, active groups trigger the polymerization of vinyl groups, which generate new active sites during chain growth, thereby perpetuating the initiation of vinyl polymerization. By employing free-radical polymerization to synthesize hyperbranched polymers, a broader range of monomers can be incorporated, and branched polymers with larger molecular weights are obtainable due to specific interactions between vinyl and active group initiation points. However, the occurrence of gels during the reaction process is a common drawback of free-radical polymerization, resulting in a low monomer conversion rate, a broad molecular weight distribution of the synthesized polymers, and the inability to produce polymers with a fixed molecular weight.

**Figure 14:**
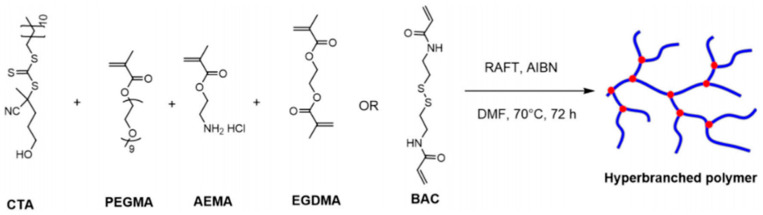
Free-radical polymerization to form hyperbranched polymers.

Marasini et al. [39] synthesized hyperbranched polymers by employing *N*,*N*-dimethylformamide (DMF) as the solvent via a free-radical polymerization process. In this reaction, 4-cyano-4((dodecylsulfonylthiocarbonyl)sulfanyl)pentanol (CTA), polyethylene glycol methyl methacrylate (PEGMA), 2-aminoethyl methacrylate hydrochloride (AEMA), and ethylene glycol dimethacrylate (EGDMA) or *N*,*N*-di(acryloyl)hydrazine (BAC) were utilized as monomers, with 2,2′-azobis(2-methylpropionitrile) (AIBN) serving as the initiator. The reaction pathway is shown in Figure 14.

The synthesis of ultra-high-molecular-weight polyacrylamide (UMPAM) is also a process of free-radical polymerization. Li Qingtao and his colleagues from the Daqing Petroleum Institute [40] successfully prepared UMPAM with a molecular weight of 20 million, utilizing a comprehensive initiator system composed of redox initiators, *N*,*N*-double-bond-containing initiators, and chain transfer agents.

Moreover, Cheng Jiecheng and his colleagues from the Dalian University of Technology [41] synthesized UMPAM with a molecular weight of 25–30 million, employing a bifunctional peroxide initiator system.

## 3. Application of Branched Polymers

Polymers with highly branched structures and modified end groups, such as dendritic polymers and hyperbranched macromolecules, display exceptional solubility, fluidity, temperature resistance, salt resistance, shear resistance, and viscosity-increasing ability. Consequently, they have emerged as a prominent focus in polymer material research. In the petrochemical industry, these branched polymers have been utilized as oil displacement agents, demulsifiers, viscosity reducers for thick oil, shale inhibitors, and sealing agents, among other applications. Their wide-ranging application prospects continue to expand to this day.

### 3.1. Oil-Displacing Agents

Polymers are a frequently employed oil displacement agent that enhances oil recovery by elevating the viscosity of the injected fluid, optimizing the oil–water ratio, and expanding the displaced fluid’s sweep volume [42]. However, during the mixing and pumping processes, the molecular chains of linear polymer solutions generally undergo shear degradation, leading to a reduction in molecular weight and solution viscosity, significantly curtailing the migration effectiveness of the polymer. Furthermore, conditions such as high temperature and salinity can exacerbate the degradation of the polymer. In contrast, the network structure of branched polymers significantly mitigates the impact of shear on polymer molecular chains, conferring a considerable advantage in polymer flooding under various extreme conditions. Consequently, branched polymers are extensively employed as oil displacement agents.

**Figure 15:**
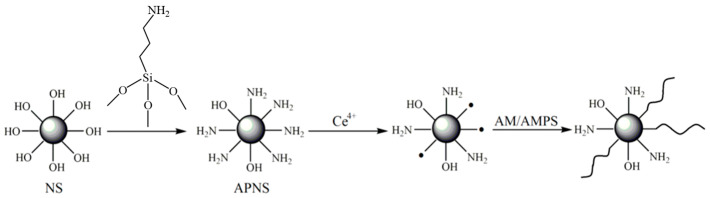
Polymer synthetic procedure.

**Figure 16:**
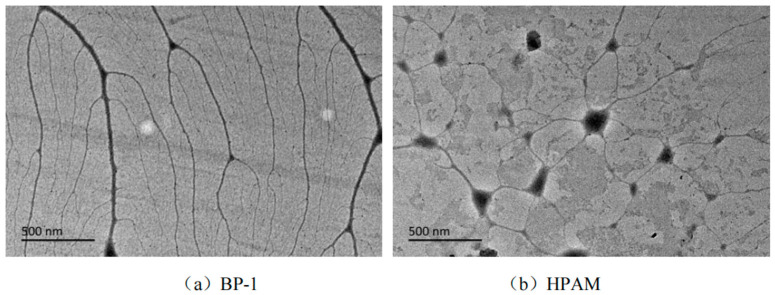
TEM micrographs of the polymers in deionized water.

Modified nano-silica particles with different molecular weights were used as cores by Lv Fei from the China University of Petroleum (East China) [43]. Cerium ammonium nitrate was employed as the initiator, while acrylamide and 2-acrylamido-2-methylpropane sulfonic acid served as monomers. Via experiments, free-radical polymerization was employed to optimize the synthetic conditions of the branched polymers and select the samples with the best performance. Consequently, two branched polymers, BP-1 and BP-2, were synthesized under optimal conditions. The synthesis steps of the polymer are illustrated in Figure 15, while Figure 16 presents TEM micrographs of the polymer in deionized water. Compared with the linear polymer HPAM, BP-1 and BP-2 displayed stable three-dimensional highly branched spatial network structures and anti-cationic compressibility, which ascribed them outstanding salt resistance. Core displacement experiments were conducted on the solutions of the branched polymers BP-1 and BP-2, as well as the linear polymer HPAM. The physical simulation oil displacement capabilities of the three polymers (BP-1, BP-2, and HPAM) were investigated at 80 °C. The results revealed that the recovery rates after the injection of BP-1, BP-2, and HPAM increased by 18.9%, 16.5%, and 11.4%, respectively, compared with water flooding. The micro oil displacement performance test results of the three polymer solutions demonstrated that BP-1 exhibited the highest final recovery rate of 67.0% (BP-2 and HPAM had final recovery rates of 63.6% and 56.8%, respectively). This indicates that the oil displacement performance of branched polymers is significantly better than that of linear polymers. Furthermore, the author suggests reinforcing research on the dissolution rate of branched polymers. Considering the synthesis cost, the branched polymer BP-1 with polyethylene imine as the core demonstrates stronger applicability.

Polymers with core–shell structures were prepared by Gou Rui from Southwest Petroleum University [44] using PAMAM hybrid carbon-nanotube-core monomers, which were copolymerized with AM/AA/modified Pingjia. The polymer solutions were evaluated for temperature resistance and shear ability. The results demonstrated that at 70 °C, the viscosities of the branched polymer solutions with concentrations of 2000 mg/L and 3000 mg/L were 44.7 mPa·s and 76.2 mPa·s, respectively. When the temperature was elevated to 90 °C, the viscosities decreased to 28.5 mPa·s and 61.3 mPa·s, respectively, which were higher than the viscosity of ordinary linear polymer HPAM under the same conditions. This suggests that the branched polymer possesses excellent temperature resistance. The polymer solution was subjected to shear for 15 s at speeds of 3600 r/min, 7200 r/min, and 11,000 r/min, with viscosity retention rates of 53%, 45%, and 43%, respectively, significantly surpassing HPAM, indicating that the branched polymer has robust shear resistance. The branched polymer exhibited an oil displacement efficiency of 45%, which is higher than that of ordinary linear polymers. The author posits that compared with other branched polymers, the incorporation of hybrid multi-walled carbon nanotubes endows the branched polymer with a more regular structure, which can potentially enhance the long-term stability of the polymer.

**Figure 17:**
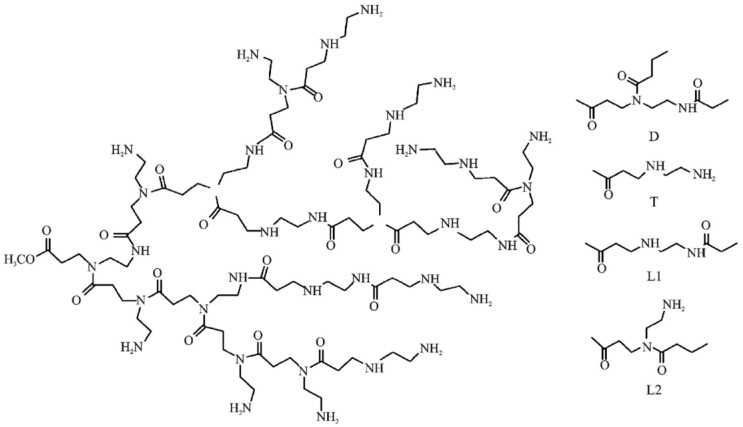
Schematic diagram of skeleton monomer structure.

**Figure 18:**
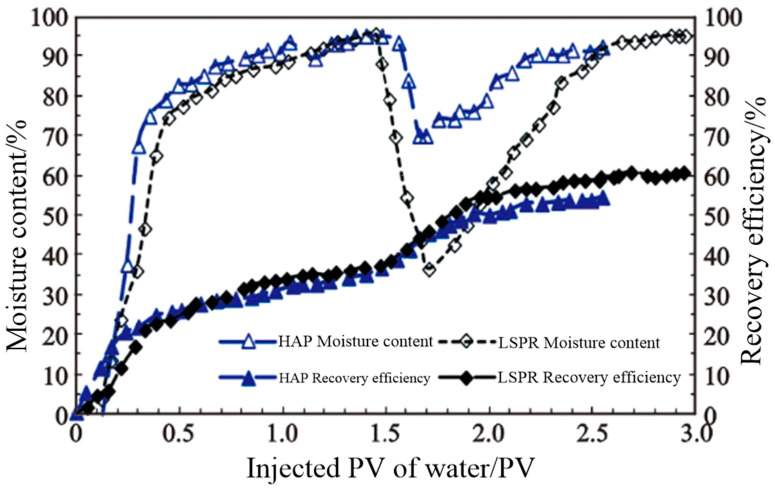
Change curve of water content and oil recovery.

Hu et al. [45] developed and synthesized a long-term shear-resistant polymer, LSRP, to address the practical requirements of offshore oil fields and enhance the temperature and salt resistance of polymers in complex oil reservoir environments. The schematic diagram in Figure 17 illustrates the monomer structure of the polymer, wherein D and T represent regular repeating units characterized by branched structures. Meanwhile, L1 and L2 denote linear repeating units with irregular structures. This polymer employs polyamine methyl acrylate as its polymerization precursor and possesses a hyperbranched structure, categorizing it as a polyamide amine monomer. A core oil displacement experiment was conducted, with the experimental outcomes presented in Table 1 and Figure 18. The data reveal that under identical crude oil viscosity and temperature conditions, HAP flooding can enhance oil recovery by approximately 6%. Furthermore, despite an increase in crude oil viscosity, LSRP flooding can significantly improve oil recovery. To validate the effectiveness of the polymer, on-site experiments were conducted in the Bai21 well area of Xinjiang, which features pronounced interlayer and intra-layer heterogeneity, severe water coning, water channeling, and water fingering. During the experiment, a smooth injection of 30 tons of LSRP dry powder was achieved, followed by a steady increase in the injection well pressure without any obstructions or abrupt pressure spikes. Moreover, no issues with solubility or foaming were encountered in the preparation equipment, indicating that the polymer effectively impedes flow resistance within oil reservoirs and exhibits excellent shear resistance.

In response to the challenges of inadequate recovery, enhanced reservoir heterogeneity, water injection fingering, and severe water breakthrough in the H area of the Xinjiang Oilfield following the implementation of special water injection treatment, Tang Zhijuan of Southwest Petroleum University [46] proposed the utilization of a sewage-based hyperbranched polymer to enhance the oil displacement efficiency in the H area. The author selected three distinct viscosity hyperbranched polymers, namely, HBP-P1, HBP-P2, and HBP-P3, and prepared various concentrations using oilfield-produced water to examine their solution properties. The findings indicated that the sewage-based hyperbranched polymer solution exhibited favorable viscosity-increasing properties. At a shear rate of 10.03 s^−1^ and a temperature of 43 °C, the viscosity of the polymer HBP-P3 with a concentration of 2500 mg/L reached 235.31 mPa·s. It demonstrated good temperature resistance, with viscosity retention rates exceeding 45%, and the higher the concentration, the better the temperature resistance. Subsequently, an indoor physical modeling experiment was conducted to investigate the oil displacement performance of the sewage-based hyperbranched polymer. The results revealed that the sewage-based hyperbranched polymer could effectively enhance the oil recovery rate. The displacement efficiency of the hyperbranched polymer exhibited an initial increase followed by a decrease as the injection rate increased. In a core with a gas permeability of 700 mD and a water permeability of 300 mD, the optimal injection rate was 0.3 mL/min when the HBP-P2 concentration was 1500 mg/L. A larger injected slug resulted in a higher oil displacement efficiency for the polymer. Increased core permeability led to an improved oil displacement efficiency of the hyperbranched polymer. The oil displacement efficiency of the sewage-based hyperbranched polymer was influenced by the size of the molecular coil. With increasing viscosity, the size of the molecular coil was enlarged, and the oil displacement efficiency initially increased before decreasing. The optimal oil displacement efficiency was achieved at a viscosity of 150 mPa·s. The HBP-P3 concentration of 1500 mg/L enhanced the oil recovery rate by 9.08% in the 1000-5-1 core.

### 3.2. Crude Oil Demulsifier

With the progression of extraction activities, the majority of extracted crude oil exists as oil-in-water (O/W) emulsions. Consequently, it is crucial to develop effective methods for the demulsification and dehydration of crude oil [47]. The majority of crude oil demulsifiers are amphiphilic compounds that can adsorb at the oil–water interface, reduce interfacial tension, enhance flocculation and aggregation, and facilitate phase separation. However, these demulsifiers often possess high costs, intricate modifications, substantial addition quantities, gradual startup effects, and limited reservoir adaptability. Due to their distinctive branched structure and modifiable terminal functional groups [48], branched polymers hold potential as demulsifiers.

**Figure 19:**
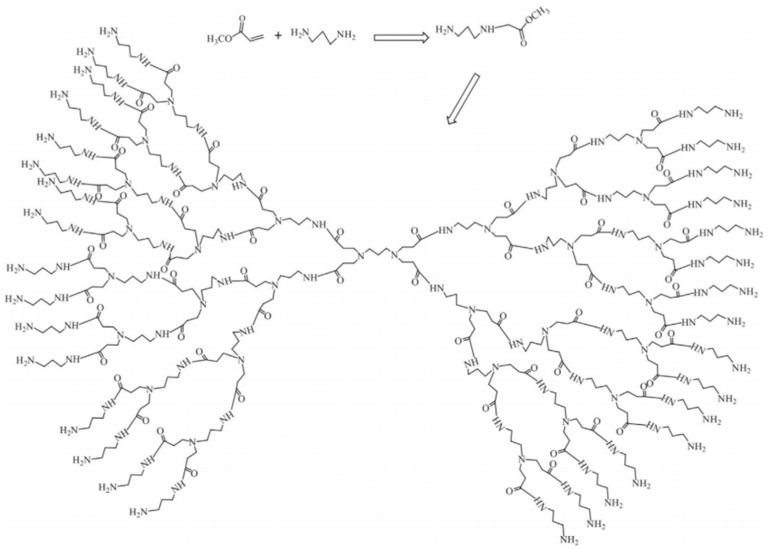
Synthetic route of H-PAMAM.

Zhang et al. [49] synthesized hyperbranched polyamine (H-PAMAM), possessing remarkable demulsification capabilities, via a “one-pot multi-step” method using methyl acrylate and ethylenediamine as synthetic materials. Figure 19 shows the synthetic route of H-PAMAM. Characterized by its hyperbranched topological structure and abundant amino groups, H-PAMAM demonstrates rapid action. The demulsification equilibrium can be achieved within merely 30 min, accompanied by a remarkable degreasing rate of 91%. Moreover, the product obtained via this method necessitates no purification, thereby reducing the overall synthesis cost.

Prepared by Feng et al. [50], hyperbranched polymers (PDBMs) with a diethyl dihydroxymethylmalonate core were synthesized via a straightforward “one pot” reaction process. The structure and thermal stability of these polymers were investigated using 1H NMR, FT IR, and TG DTG techniques. A systematic investigation was conducted on the effects of the demulsifier dosage, settling time, and mineralization degree on the demulsification performance of an oil-in-water (O/W) emulsion at a temperature of 25 °C. Following the addition of 100 mg/L of PDBM and standing at 25 °C for 60 min, the transmittance of separated water (TSW) reached a value of 91.4%. To further explore the underlying mechanism, structurally similar polymers (PDM) were synthesized using the same method. The findings suggest that the hydroxyl groups in the core region of PDBM play a vital role in the demulsification process of O/W emulsions.

To minimize the synthesis steps and production costs of hyperbranched polymers, Yan et al. [51] employed hyperbranched polyethylene imine (HPEI) as the core and a fatty-acid-based polymer (Cn) as the shell to synthesize a novel core–shell amphiphilic polymer, HPEI-g-Cn, via a one-step method. Cn typically consists of 10, 12, 14, 16, and 18 saturated fatty acids. The findings indicate that in the presence of HPEI-g-Cn, the oil–water emulsion can be completely separated into two phases within 40 min, achieving an oil removal rate of over 99.9%. After demulsification, HPEI-g-Cn is uniformly and densely distributed at the oil–water interface, facilitating subsequent recovery, enhancing environmental friendliness, and reducing application costs.

### 3.3. Heavy Oil Viscosity Reducer

The abundant geological reserves of heavy oil worldwide render it an essential petroleum resource. However, the presence of resins and asphaltenes significantly increases the viscosity of crude oil, hindering its flow and leading to a low recovery rate of heavy oil. Consequently, the discovery of effective methods to reduce heavy oil viscosity is a prerequisite for enhancing crude oil recovery. The employment of chemical viscosity reducers, due to their efficiency and cost-effectiveness, is a widely adopted technique. Despite the majority of reported viscous oil viscosity reducers being linear polymers, their effectiveness in reducing viscous oil viscosity is limited. Consequently, researchers have begun exploring the use of hyperbranched polymers as viscous oil viscosity reducers.

Yan Zhenhu, a researcher from Shandong University [52], conducted comprehensive research on heavy oil viscosity reducers for the high-viscosity heavy oil in the Bohai Sea. He synthesized two types of dendritic polyamide amines (G1.0, G2.0, D2.0, and T2.0) and explored their viscosity-reducing effects and mechanisms. Firstly, a series of dendritic polyamide amines were synthesized, and G1.0 and G2.0 were electrostatically neutralized and capped with different organic acids (acetic acid, butyric acid, caproic acid, and oleic acid) and acrylamide morpholine, introducing hydrophobic alkyl chains. Subsequently, these modified polyamides were employed to decrease the viscosity of heavy oil. The results indicated that the heavy oil viscosity reducers regulated with caproic acid (G1.0 and G2.0) exhibited outstanding performance, with a viscosity reduction rate of up to 96% at a low concentration of 200 mg/L, and stability analysis and testing revealed that the lotion system formed by the heavy oil was less stable than the G2.0 regulated with caproic acid. Moreover, polyamides with high branching degrees, such as D2.0 and T2.0, demonstrated good viscosity reduction effects for heavy oil without the addition of organic acids, reaching a viscosity reduction rate of 95% at a concentration of 200 mg/L.

**Figure 20:**
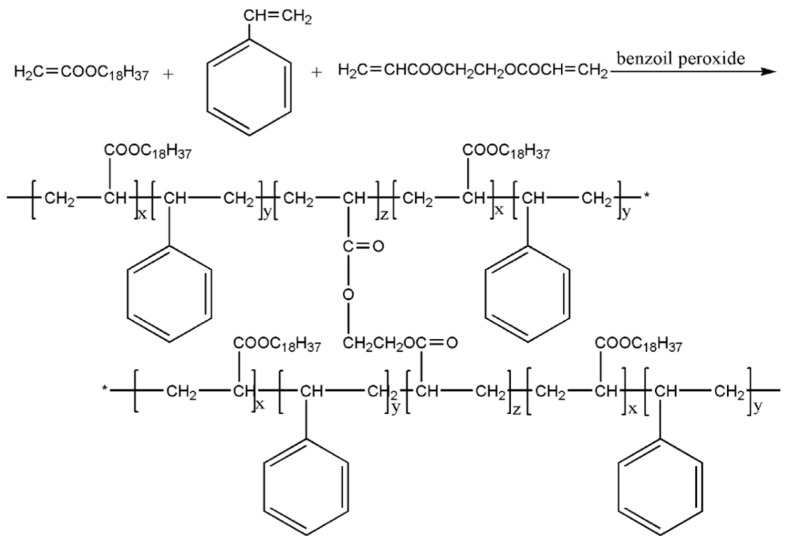
Preparation of viscosity reducer (“*” marked structural formulas are connected).

Polymers have been synthesized by Zheng et al. [53] for the viscosity reduction of heavy oil, utilizing octadecyl methacrylate, styrene, and ethylene glycol dimethacrylate as raw materials. Figure 20 shows the preparation of the viscosity reduction process. The results of the viscosity reduction experiment demonstrate that a reduction rate of over 60% can be achieved when the concentration of heavy oil is 800 ppm. By analyzing the effects of varying concentrations of viscosity-reducing agents on the settlement point, settlement amount, and asphalt particle size, it was determined that this hyperbranched polymer viscosity reducer enhances the settlement point of asphalt and markedly reduces the settlement amount and average particle size of asphalt. The formation of asphaltene is inhibited by altering the polarity of asphaltene molecules, generating hydrogen bonds, dispersing asphaltene molecules, adsorbing or embedding asphaltene molecular fragments, and other approaches. This polymer exhibits excellent dispersion and inhibition effects on asphaltene precipitation. The synthesis of this hyperbranched polymer viscosity reducer significantly reduces the cost while ensuring high performance.

### 3.4. Shale Inhibitor

The presence of significant quantities of clay minerals (mainly sodium-based bentonite) and hard, brittle minerals in shale makes it susceptible to hydration and expansion in contact with water-based drilling fluids during the drilling process. This can ultimately lead to wellbore instability and collapse [54]. To mitigate the detrimental effects of sodium-based bentonite, domestic and international researchers have developed a range of polymer shale inhibitors. However, due to the limited number of inhibitory functional groups and the linear molecular structure, the adsorption of these linear polymer shale inhibitors on sodium-based bentonite is often uneven and irregular, with relatively few adsorption sites. Under high-temperature and high-speed turbulent conditions, the adsorption efficacy of linear polymer shale inhibitors on sodium-based bentonite is further compromised. In contrast, branched polymers possess numerous branched structures, with many end groups at the terminal of the molecular chain. By incorporating multiple amino groups into these end groups, their adsorption on clay can be enhanced, dehydrating the clay crystal layer and reducing the swelling force. Concurrently, the polyamine groups along the molecular chain can anchor the clay flakes, disrupting their hydration structure and thereby optimizing the inhibitory effect of amine inhibitors on shale.

**Figure 21:**
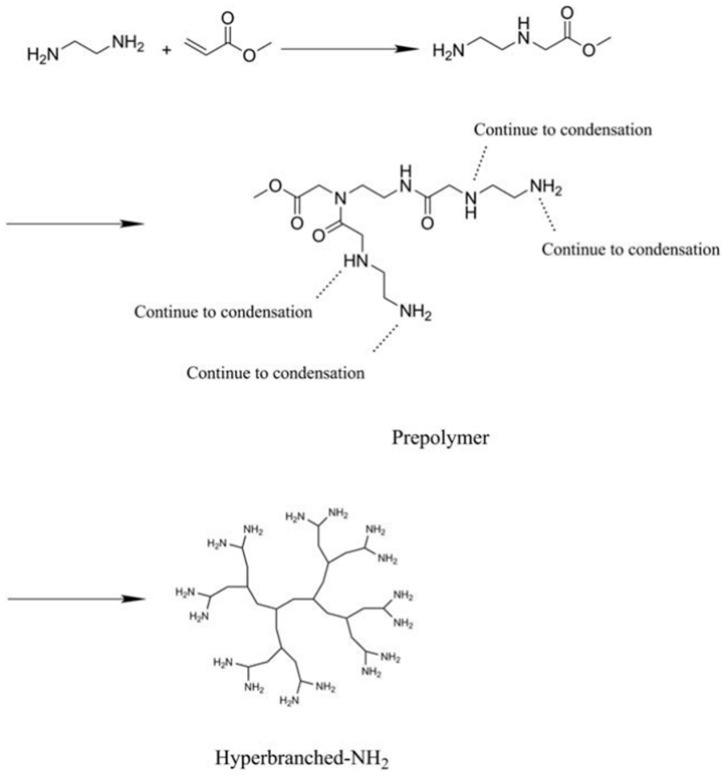
Synthetic scheme of HBP-NH_2_.

Bai et al. [55] synthesized an amine-terminated hyperbranched polymer (HBP-NH_2_), a water-based drilling fluid inhibitor prepared via the condensation polymerization of a diamine AB2 monomer. Figure 21 shows the synthetic scheme of HBP-NH_2_. To optimize the polymerization conditions, a linear expansion rate was employed as the performance evaluation criterion. When the molar ratio of amine to ester was 1:1, HBP-NH2 exhibited an excellent inhibitory performance at 100 °C, 120 °C, and 140 °C for 2 h, respectively. Furthermore, thermogravimetric analysis revealed that the synthesized HBP-NH_2_ exhibited good temperature resistance prior to the drilling fluid temperature reaching 200 °C. The linear expansion rate of HBP-NH_2_ at various concentrations was also tested, and the results indicated that the linear expansion rate was only 11.42%, which was lower than that of other traditional inhibitors. Compared with linear inhibitors, the adsorption and encapsulation of HBP-NH_2_ in sodium-based bentonite were more uniform due to HBP-NH_2_’s highly branched structure and numerous terminal primary amine groups. ESEM analysis showed that HBP-NH_2_ possesses a typical layered structure and rough surface, which can effectively achieve adsorption and encapsulation. Zeta probe analysis demonstrated that protonated terminal amine groups can adsorb on the negatively charged surfaces of sodium-based bentonite particles, inhibiting their dispersion tendency. Lastly, the inhibitory effect of HBP-NH_2_ on the dispersion of shale cuttings was confirmed via shale-rolling experiments. When the concentration of HBP-NH_2_ was 5 wt%, the rolling recovery rate of shale cuttings was 68.54 wt%.

Ferreira et al. [56] synthesized a non-ionic active shale inhibitor, hydrophobic hyperbranched polyglycerol, using glycerol, dimethyl carbonate, and trimethylpropane as raw materials. By incorporating KCl, the hydrophobic hyperbranched polyglycerol demonstrated superior performance compared with unmodified hyperbranched polyglycerol, with a complete cutting recovery rate of approximately 80%. The proposed inhibition mechanism suggests the formation of a complex between hydrophobic hyperbranched polyglycerol and K+ ions, which permeates into the interlayer spacing of clay layers to minimize shale–water interactions and extract water molecules from the clay corridor. Furthermore, the aggregates formed by amphiphilic structures constrict the pore throats of clay minerals, rendering it more challenging for water molecules to permeate.

### 3.5. Plugging Agent

Potential challenges in the development of low-permeability oilfields have necessitated the implementation of hydraulic fracturing, which serves as a crucial strategy for enhancing production. However, the continuous progression of this technique has led to an annual increase in the water content in oil wells, thereby depleting the crude oil within the original fracture control range. Furthermore, the reservoir depth of 2500–3000 m is characterized by severe environmental conditions, such as high temperatures and salinity. To overcome these challenges and facilitate successful steering refracturing in this complex reservoir environment, stringent temperature and salt resistance requirements must be met for the employed gels.

In light of this scenario, Tan Long from Southwest Petroleum University [57] opted for the branched polymer AP-P5 and the composite cross-linking agent VT-16 to construct a gel-based cross-linking system after meticulously examining the properties and traits of various polymer gels. The optimized formula for the cross-linking system derived from in-house experiments was 0.2% branched polymer AP-P5+4% composite crosslinking agent A+0.4% composite crosslinking agent B. A comprehensive investigation of the fundamental properties and primary application characteristics of the gel–water shutoff agent was conducted. The findings suggested that the gel strength remained at the F level for an extended period after gel formation under the conditions of a 90 °C temperature and a 2 × 10^4^ mg/L salinity. Ultimately, the primary service performance of the sealing agent was evaluated via the application of split-core and sand-filled pipe columns. The gel plug demonstrated excellent injectability. The gel strength exceeded the fracturing gradient to 44 MPa/m, achieving a plugging rate of over 92%. The breakthrough pressure gradient of the gel plug after gelling in the large fracture channel simulated by the sand-filled pipe string reached 55 MPa, with the plugging rate surpassing 95%, marginally surpassing the fracturing core. These results corroborate the contention that the gel-plugging agent exhibits a more potent plugging effect in large fractures and can effectively cater to the requirements of on-site construction.

## 4. Summaries

### 4.1. Conclusions

With the ongoing advancements in tertiary oil recovery technology and the increasing depths of reservoir exploration and development, the reservoir environment is becoming progressively more challenging. Conventional linear polymers, plagued by inadequate temperature resistance and shear resistance, are no longer suitable for the current complex requirements of oil reservoir environments. In contrast, branched polymers exhibit superior properties such as high fluidity, temperature resistance, shear resistance, and ease of modification. Both dendritic polymers and hyperbranched macromolecules have broad applications in the petrochemical industry.

(1) The divergent synthetic method is currently the primary strategy for the preparation of dendritic polymers, involving an initial core containing reactive functional groups that initiate polymerization in a stepwise manner from the interior to the exterior. However, the increase in reaction complexity leads to a higher propensity for defects in the dendritic molecular structure. The convergent synthetic method offers superior control over the dendritic polymer structure, albeit with a relatively low yield, which is currently limiting its broader application. The complexity of the reaction process and the associated high synthesis costs have precluded the widespread adoption of the divergent–convergent co-use method.
(2) Polymers with hyperbranched structures are primarily synthesized using the condensation reaction method, which benefits from well-established synthetic conditions, straightforward operation procedures, and relatively low production costs. As research into these materials continues to advance, additional methods for synthesizing hyperbranched macromolecules have emerged, including self-condensation vinyl polymerization, cross-coupling reactions, coupling monomer approaches, and the Huisgen reaction. Each of these techniques possesses its own merits and drawbacks, and it is recommended that the most appropriate synthetic method be selected based on the specific requirements of industrial applications.
(3) Polymers with a branched structure, under complex conditions such as high temperatures and salinity in oil fields, can offer ample sites for the grafting of various groups. Loaded with diverse chemical groups, these polymers can exhibit exceptional properties, including temperature resistance, shear resistance, and salt resistance. In the realm of the petrochemical industry, these branched polymers are not only applicable in the aforementioned areas but can also be employed in fields related to scale inhibitors, fracturing fluid thickeners, water-blocking agents, and drilling fluids. Their application scope is continuously expanding.

### 4.2. Future Prospects

(1) Currently, numerous branched polymers in the field of petrochemical research are still at the stage of laboratory synthesis and evaluation. Further field tests are required to validate the feasibility of industrial applications for these branched polymers. For instance, addressing the urgent issues of synthesis costs and reaction efficiency is crucial for their widespread implementation.
(2) Considering the synthesis cost and efficacy of various branched polymers, it is generally advisable to utilize hyperbranched polymers instead of dendrimers in practical industrial applications unless there are stringent requirements for polymer performance.
(3) If the synthesis of hyperbranched polyamide esters or similar ester polymers is required, it is recommended to employ polycondensation synthesis. Ring-opening polymerization is commonly utilized for synthesizing hyperbranched polymers with high molecular weights and amphiphilicity, and the resulting polymers do not necessitate the removal of small-molecule compounds. Free-radical polymerization is suitable for synthesizing products using olefin monomers containing unsaturated double bonds.

## Figures and Tables

**Table 1:** Comparison of oil displacement effects of different polymer solutions.

Polymer Types	Permeability/10^−3^ μm^2^	Water Drive Recovery Rate/%	Polymer Flooding Recovery Rate/%	Improvement Rate/%	Notes
HAP	2.025	35.11	54.56	16.45	Crude oil viscosity 70 mPa·s; 65 °C
LSRP	2.248	37.71	60.17	22.46	
LSRP	2.573	20.51	45.58	25.07	Crude oil viscosity 300 mPa·s; 65 °C

## Data Availability

Not applicable.
